# Effectiveness of COVID-19 vaccines against SARS-CoV-2 variants of concern: a systematic review and meta-analysis

**DOI:** 10.1186/s12916-022-02397-y

**Published:** 2022-05-23

**Authors:** Baoqi Zeng, Le Gao, Qingxin Zhou, Kai Yu, Feng Sun

**Affiliations:** 1 Department of Science and Education, Peking University Binhai Hospital, Tianjin, China; 2 Department of Pharmacology and Pharmacy, The University of Hong Kong, Hong Kong, China; 3 Tianjin Centers for Disease Control and Prevention, Tianjin, China; 4 Department of Epidemiology and Biostatistics, School of Public Health, Peking University Health Science Centre, Beijing, China

**Keywords:** SARS-CoV-2, COVID-19, Variants of concern, Systematic review, Vaccine effectiveness

## Abstract

**Background:**

It was urgent and necessary to synthesize the evidence for vaccine effectiveness (VE) against SARS-CoV-2 variants of concern (VOC). We conducted a systematic review and meta-analysis to provide a comprehensive overview of the effectiveness profile of COVID-19 vaccines against VOC.

**Methods:**

Published randomized controlled trials (RCTs), cohort studies, and case-control studies that evaluated the VE against VOC (Alpha, Beta, Gamma, Delta, or Omicron) were searched until 4 March 2022. Pooled estimates and 95% confidence intervals (CIs) were calculated using random-effects meta-analysis. VE was defined as (1-estimate).

**Results:**

Eleven RCTs (161,388 participants), 20 cohort studies (52,782,321 participants), and 26 case-control studies (2,584,732 cases) were included. Eleven COVID-19 vaccines (mRNA-1273, BNT162b2, ChAdOx1, Ad26.COV2.S, NVX-CoV2373, BBV152, CoronaVac, BBIBP-CorV, SCB-2019, CVnCoV, and HB02) were included in this analysis. Full vaccination was effective against Alpha, Beta, Gamma, Delta, and Omicron variants, with VE of 88.0% (95% CI, 83.0–91.5), 73.0% (95% CI, 64.3–79.5), 63.0% (95% CI, 47.9–73.7), 77.8% (95% CI, 72.7–82.0), and 55.9% (95% CI, 40.9–67.0), respectively. Booster vaccination was more effective against Delta and Omicron variants, with VE of 95.5% (95% CI, 94.2–96.5) and 80.8% (95% CI, 58.6–91.1), respectively. mRNA vaccines (mRNA-1273/BNT162b2) seemed to have higher VE against VOC over others; significant interactions (*p*_interaction_ < 0.10) were observed between VE and vaccine type (mRNA vaccines vs. not mRNA vaccines).

**Conclusions:**

Full vaccination of COVID-19 vaccines is highly effective against Alpha variant, and moderate effective against Beta, Gamma, and Delta variants. Booster vaccination is more effective against Delta and Omicron variants. mRNA vaccines seem to have higher VE against Alpha, Beta, Gamma, and Delta variants over others.

**Supplementary Information:**

The online version contains supplementary material available at 10.1186/s12916-022-02397-y.

## Background

Since emerging of coronavirus disease 2019 (COVID-19) caused by a novel severe acute respiratory syndrome coronavirus 2 (SARS-CoV-2) in December 2019, more than 452 million cases and 6.0 million deaths have been documented worldwide as of 12 March 2022 [1]. COVID-19 vaccines have been rapidly developed and proved to be highly effective in multiple randomized clinical trials (RCTs) [2–5] and observational studies [6–8]. As of 5 March 2022, more than 10 billion vaccine doses have been administered all over the world, but around 150 thousand new cases are diagnosed each day [1]. Most current vaccines used SARS-CoV-2 spike protein as the key antigenic target based on the originally identified Wuhan lineage virus [9]. The B.1.1.7 (Alpha) variant was first identified from genomic sequencing of samples obtained from COVID-19 patients which accounted for an expanding proportion of cases in England in late 2020 [10]. Subsequently, the emergence of the B.1.351 (Beta) variant in South Africa and the P.1 (Gamma) variant in Brazil increased the COVID-19 pandemic. In December 2020, a novel SARS-CoV-2 variant, the B.1.617.2 (Delta) variant was first detected in India, causing a sharp increase in COVID-19 cases and deaths in India and surrounding countries [11]. Recently, the B.1.1.529 (Omicron) variant emerged in December 2021 contains more than 30 mutations in the spike protein, raising concerns for naturally acquired or vaccinated population [12]. The emerging Alpha, Beta, Gamma, Delta, and Omicron variants were classified as variants of concern (VOC), which were associated with the transmission increasing, more severe disease situation (e.g., increased hospitalizations or deaths), significant reduction in neutralization by antibodies generated during previous infection or vaccination, reduced effectiveness of treatments or vaccines, or diagnostic detection failures [13–18]. The importance of vaccination programs and efficient public health measures will be increased if VOC have increased transmissibility or virulence [19]. It was urgent and necessary to synthesize evidence of the vaccine effectiveness (VE) of COVID-19 vaccines against VOC. To our knowledge, there are some studies evaluating the VE of COVID-19 vaccines against VOC [20–23]. Some relevant systematic review or meta-analysis about COVID-19 vaccines against Delta variant have been published to date [24–26], which did not include many recent studies as the most recent retrieval date was October 2021. Therefore, to gain insight in the VE of COVID-19 vaccines against five kinds of VOC, we conducted a comprehensive systematic review and meta-analysis including both RCTs and observational studies. This review of the VE of COVID-19 vaccines against VOC will support global response on public health measures and vaccination programs timely and evidence based.

## Methods

### Data sources and searches

We conducted this systematic review according to the Preferred Reporting Items for Systematic reviews and Meta-Analyses (PRISMA) guidelines [27]; the protocol was registered on PROSPERO (CRD42021273986). We searched for literature published on PubMed, Embase, Cochrane Library, and the ClinicalTrials.gov website on or before 4 March 2022. Keywords including “COVID-19,” “SARS-CoV-2,” “vaccine,” and “variant” were used to search; the detailed search strategy was shown in the Additional file 1 (Appendix S1). Additionally, we identified references by searching the reference lists of included studies and relevant reviews.

### Selection of studies

We included randomized controlled trials (RCTs), cohort studies, and case-control studies that evaluated the efficacy or effectiveness of COVID-19 vaccines against VOC including B.1.1.7 (Alpha), B.1.351 (Beta), P.1 (Gamma), B.1.617.2 (Delta), and B.1.1.529 (Omicron). Studies enrolling general population or special populations (e.g., healthcare workers) aged 12 years or older were included. For studies that only reported VE against SARS-CoV-2 infections (without subgroup analysis of VOC), but the specific VOC accounted for 50% or more among positive tests, they were also included in the analysis. We excluded study protocols, editorials, comments, reviews, news, case reports, conference abstracts, animal studies, in vivo experiments, and analysis of antibody neutralization. Searches were limited to English articles. The primary outcome was the VE of full vaccination against VOC; the studies which only reported the VE of partial vaccination were excluded.

### Data extraction

Two authors reviewed titles and abstracts independently to identify eligible studies that met pre-specified inclusion criteria and extracted data. When consensus was lacking, a third reviewer was consulted. The journal name, study type, study location, vaccine information, number of participants, characteristics of subjects, and outcomes were extracted from eligible studies. We extracted SARS-CoV-2 infection information if results on both SARS-CoV-2 infection and symptomatic infection were reported. The adjusted VE or estimates of effect size (relative risks, incidence rate ratios, or odds ratios) with corresponding 95% confidence intervals (CIs) were extracted with priority. The risk of bias of RCTs was assessed using the Cochrane Collaboration’s tool [28, 29]. The risk of bias of cohort and case-control studies was assessed using the Newcastle-Ottawa scale (NOS) [30].The NOS contains 8 categories relating to methodological quality, with a maximum of 9 points. A total score of 7–9 points is considered of good quality, while a score of 4–6 points of moderate quality, and a score of 1–3 points of low quality. Two investigators reviewed the studies and judged the risk of bias.

### Statistical analysis

Pooled estimates and 95% CIs were calculated using DerSimonian and Laird random-effects meta-analysis [31]. Summary VE was defined as (1-pooled estimate) ×100%. We performed subgroup analysis stratifying by study design, vaccine type, participant, and publication. *P* for the difference was calculated using random-effects meta-regression, a difference between the estimates of these subgroups was considered significant if *p*_interaction_ < 0.10 [32]. Statistical heterogeneity between the studies was assessed with the *χ*^2^ test and the *I*^2^ statistics. *I*^2^ values of 25%, 50%, and 75% have been suggested to be indicators of low, moderate, and high heterogeneity, respectively [33]. All the analyses were performed with STATA 14.

## Results

### Literature search and study characteristics

This systematic literature search identified 6740 publications; after excluding duplicates and irrelevant papers, 219 published reports were evaluated in full text for eligibility (Additional file 1: Figure S1). Finally, 57 articles were included in the present systematic review [6, 7, 20–23, 34–84]. There were different study designs for included studies, 11 RCTs (161,388 participants) [20, 22, 23, 34–41], 20 cohort studies (52,782,321 participants) [6, 7, 42–59], and 26 case-control studies (2,584,732 cases) [21, 60–84]. In total, 11 COVID-19 vaccines (mRNA-1273, BNT162b2, ChAdOx1, Ad26.COV2.S, NVX-CoV2373, BBV152, CoronaVac, BBIBP-CorV, SCB-2019, CVnCoV, and HB02) and 5 VOC (Alpha, Beta, Gamma, Delta, and Omicron) were included in this study. BNT162b2, mRNA-1273, and CVnCoV are mRNA vaccines; CoronaVac, HB02, BBV152, and BBIBP-CorV are inactivated vaccines; Ad26.COV2.S and ChAdOx1 are non-replicating vector vaccines; and NVX-CoV2373 and SCB-2019 are protein subunit vaccines. Only Ad26.COV2.S is a single-dose vaccine; therefore, a one-dose regimen is regarded as full vaccination. Characteristics of individual studies are summarized in Table 1.

**Table 1:** Study characteristics and participants demographics

First author	Journal	Study design	VOC ^c^	Vaccine	Country	Population characteristics	*N*
Shinde (2021) [20]	N Engl J Med	RCT	Beta (GS)	NVX-CoV2373	South Africa	GP; age: 18–84	4,387
Madhi (2021) [22]	N Engl J Med	RCT	Beta (GS)	ChAdOx1	South Africa	GP; age: 18–65	1,467
Heath (2021) [38]	N Engl J Med	RCT	Alpha (GS)	NVX-CoV2373	UK	GP; age: 18–84	14,039
Sadoff (2022) [40]	N Engl J Med	RCT	Beta (GS)	Ad26.COV2.S	South Africa	GP; age: ≥18	4,969
Emary (2021) [23]	Lancet	RCT	Alpha (GS)	ChAdOx1	UK	GP; age: ≥18	8,534
Ella (2021) [37]	Lancet	RCT	Delta (GS)	BBV152	India	GP; age: 18–98	16,973
Thomas (2021) [41]	N Engl J Med	RCT	Beta (GS)	BNT162b2	South Africa	GP; age: ≥16	800
Clemens (2021) [35]	Nat Commun	RCT	Gamma (GS)	ChAdOx1	Brazil	GP; age: ≥18	10,416
Bravo (2022) [34]	Lancet	RCT	Gamma & Delta (GS)	SCB-2019	Five regions	GP; age: ≥18	30,174
Dunkle (2022) [36]	N Engl J Med	RCT	Alpha (GS)	NVX-CoV2373	USA and Mexico	GP; age: ≥18	29,949
Kremsner (2022) [39]	Lancet Infect Dis	RCT	Alpha & Gamma (GS)	CVnCoV	Europe/Latin America	GP; age: ≥18	39,680
Lopez Bernal_1 (2021) [21]	N Engl J Med	TNCC	Alpha & Delta (GS)	BNT162b2 or ChAdOx1	UK	GP; age: ≥16	19,109 ^b^
Abu-Raddad (2021) [60]	N Engl J Med	TNCC	Alpha & Beta (GS)	BNT162b2	Qatar	GP; age: 33 (22–40) ^a^	35,979 ^b^
Sheikh (2021) [80]	Lancet	TNCC	Alpha & Delta (GS)	BNT162b2 or ChAdOx1	UK	GP; age: ≥16	19,543 ^b^
Chemaitelly_1 (2021) [67]	Nat Med	TNCC	Alpha & Beta (GS)	mRNA-1273	Qatar	GP; age: 32 (25–39) ^a^	66,042 ^b^
Lopez Bernal_2 (2021) [76]	BMJ	TNCC	Alpha	BNT162b2	UK	Older adults; age: ≥70	3,034 ^b^
Chung (2021) [68]	BMJ	TNCC	Alpha & Beta/Gamma (GS)	BNT162b2 and mRNA-1273	Canada	GP; ≥16	324,033 ^b^
Ranzani (2021) [79]	BMJ	TNCC	Gamma	CoronaVac	Brazil	Older adults; ≥70	43,774 ^b^
Carazo (2021) [64]	Clin Infect Dis	TNCC	Alpha	BNT162b2 and mRNA-1273	Canada	HCWs; age: 18–74	901 ^b^
Li (2021) [75]	Emerg Microbes Infect	TNCC	Delta (GS)	CoronaVac and BBIBP-CorV	China	GP; age:18–59	74 ^b^
Charmet (2021) [65]	Lancet Reg Health Eur	Case-control	Alpha & Beta/Gamma	BNT162b2 and mRNA-1273	France	GP; age: ≥20	33,863 ^b^
Nasreen (2022) [77]	Nat Microbiol	TNCC	Alpha & Beta & Gamma & Delta (GS)	BNT162b2, mRNA-1273, or ChAdOx1	Canada	GP; age: ≥16	51,440 ^b^
Hitchings_1 (2021) [73]	Lancet Reg Health Am	TNCC	Gamma	CoronaVac	Brazil	HCWs; age: ≥18	418 ^b^
Tang (2021) [82]	Nat Med	TNCC	Alpha & Delta	BNT162b2 or mRNA-1273	Qatar	GP; age: 27 (12–36) ^a^	2,934 ^b^
Chemaitelly_2 (2021) [66]	N Engl J Med	TNCC	Alpha & Beta & Delta (GS)	BNT162b2	Qatar	GP; age: 31 (21–39) ^a^	113,830 ^b^
Grannis (2021) [70]	MMWR	TNCC	Delta	BNT162b2, mRNA-1273 or Ad26.COV2.S	USA	GP; age: ≥18	3,657 ^b^
Sritipsukho (2022) [81]	Emerg Microbes Infect	TNCC	Delta	CoronaVac and/or ChAdOx1	Thailand	GP; age: ≥18	1,118 ^b^
Klein (2022) [74]	MMWR	TNCC	Delta & Omicron	BNT162b2 or mRNA-1273	USA	GP; age: ≥18	3,860 ^b^
Oliveira (2022) [78]	JAMA Network Open	TNCC	Delta (GS)	BNT162b2	USA	Adolescents; 12–18	186 ^b^
Tseng (2022) [84]	Nat Med	TNCC	Delta & Omicron (GS)	mRNA-1273	USA	GP; age: ≥18	23,512 ^b^
Ferdinands (2022) [69]	MMWR	TNCC	Delta & Omicron	BNT162b2 or mRNA-1273	USA	GP; age: ≥18	18, 637 ^b^
Britton (2022) [63]	JAMA	TNCC	Delta	BNT162b2, mRNA-1273, or Ad26.COV2.S.	USA	GP; age: ≥12	329,057 ^b^
Grant (2022) [71]	Lancet Reg Health Eur	Case-control	Delta	BNT162b2 or mRNA-1273	France	GP; age: ≥20	8,644 ^b^
Andrews_1 (2022) [62]	N Engl J Med	TNCC	Delta	BNT162b2 or ChAdOx1	UK	GP; age: ≥16	1,125,257 ^b^
Andrews_2 (2022) [61]	Nat Med	TNCC	Delta (GS)	BNT162b2	UK	GP; age: ≥18	343,955 ^b^
Thiruvengadam (2021) [83]	Lancet Infect Dis	TNCC	Delta (GS)	ChAdOx1	India	GP; age: 35 (28–45) ^a^	2,766 ^b^
Hitchings_2 (2021) [72]	Nat Commun	TNCC	Gamma	ChAdOx1	Brazil	Older adults; ≥60	30,680 ^b^
Hall (2021) [6]	Lancet	PRO cohort	Alpha	BNT162b2	UK	HCWs; age: ≥18	23,324
Haas (2021) [46]	Lancet	RETRO cohort	Alpha	BNT162b2	Israel	GP; age: ≥16	6,538,911
Dagan (2021) [7]	N Engl J Med	RETRO cohort	Alpha	BNT162b2	Israel	GP; age: ≥16	1,193,236
Lumley (2021) [49]	Clin Infect Dis	RETRO cohort	Alpha (GS)	BNT162b2 and ChAdOx1	UK	HCWs; age: 39(30–50) ^a^	13,109
Williams (2021) [58]	Clin Infect Dis	RETRO cohort	Gamma (GS)	mRNA-1273	Canada	LTCH	143
Nanduri (2021) [51]	MMWR	RETRO cohort	Delta	mRNA-1273 or BNT162b2	US	Nursing home	5,965,607
Fowlkes (2021) [44]	MMWR	RETRO cohort	Delta	mRNA-1273 and BNT162b2	US	Frontline workers	2,840
Pouwels (2021) [53]	Nat Med	RETRO cohort	Alpha & Delta	BNT162b2 or ChAdOx1 or mRNA-1273	UK	GP; age: 18–64	743,526
Flacco (2021) [43]	Vaccines	RETRO cohort	Alpha	BNT162b2	Italian	GP; age: ≥18	204,840
Glatman-Freedman (2021) [45]	Emerg Infect Dis	RETRO cohort	Delta	BNT162b2	Israel	Adolescents; 12–15	601,625
Seppälä (2021) [56]	Euro Surveill	RETRO cohort	Alpha & Delta (GS)	BNT162b2 or mRNA-1273	Norway	GP; age: ≥18	18,431
Fabiani (2022) [42]	BMJ	RETRO cohort	Alpha & Delta	BNT162b2 or mRNA-1273	Italy	GP; age: ≥16	33,250,344
Risk (2022) [55]	Clin Infect Dis	RETRO cohort	Delta	BNT162b2, mRNA-1273, or Ad26.COV2.S.	USA	GP; age: ≥18	159,055
Kang (2022) [47]	Ann Intern Med	RETRO cohort	Delta (GS)	CoronaVac or HB02	China	GP; age: ≥18	10,805
Poukka (2022) [52]	Vaccine	RETRO cohort	Delta	BNT162b2 or mRNA-1273 or ChAdOx1	Finland	16–69 HCWs	427,905
Wu (2022) [59]	China CDC Wkly	RETRO cohort	Delta (GS)	BBIBP-CorV or CoronaVac	China	Close contacts; age: ≥18	1,462
Katz (2022) [48]	Vaccine	PRO cohort	Alpha (GS)	BNT162b2	Israel	HCWs; age: 45 (36–55) ^a^	1,250
Lutrick (2021) [50]	MMWR	PRO cohort	Delta	BNT162b2	USA	Adolescents; 12–17	243
Reis (2021) [54]	N Engl J Med	RETRO cohort	Delta	BNT162b2	Israel	Adolescents; 12–18	188,708
Tartof (2021) [57]	Lancet	RETRO cohort	Delta (GS)	mRNA-1273	USA	GP; age: ≥12	3,436,957

Abbreviations: *VOC* variants of concern, *HCWs* healthcare workers, *TNCC* test-negative case-control, *LTCH* long-term care homes, *GP* general population, *RCT* randomized controlled trial, *GS* genomic sequencing, *N* number of participants, *PRO* prospective, *RETRO* retrospective

^a^Median age (interquartile range)

^b^Cases

^c^VOC were identified by genomic sequencing (GS) or variant circulation dominance

### Risk of Bias

All the RCTs were assessed as some concerns for overall risk-of-bias judgment. Fifteen of 20 cohort studies were judged as good quality, and the remaining 5 studies were moderate quality. For 26 case-control studies, 22 were considered as good quality and 4 were moderate quality. The detailed risk of bias assessment is available in Additional file 1 (Tables S1–S3).

### Vaccine effectiveness of COVID-19 vaccines against B.1.1.7 (Alpha) variant

**Fig. 1:**
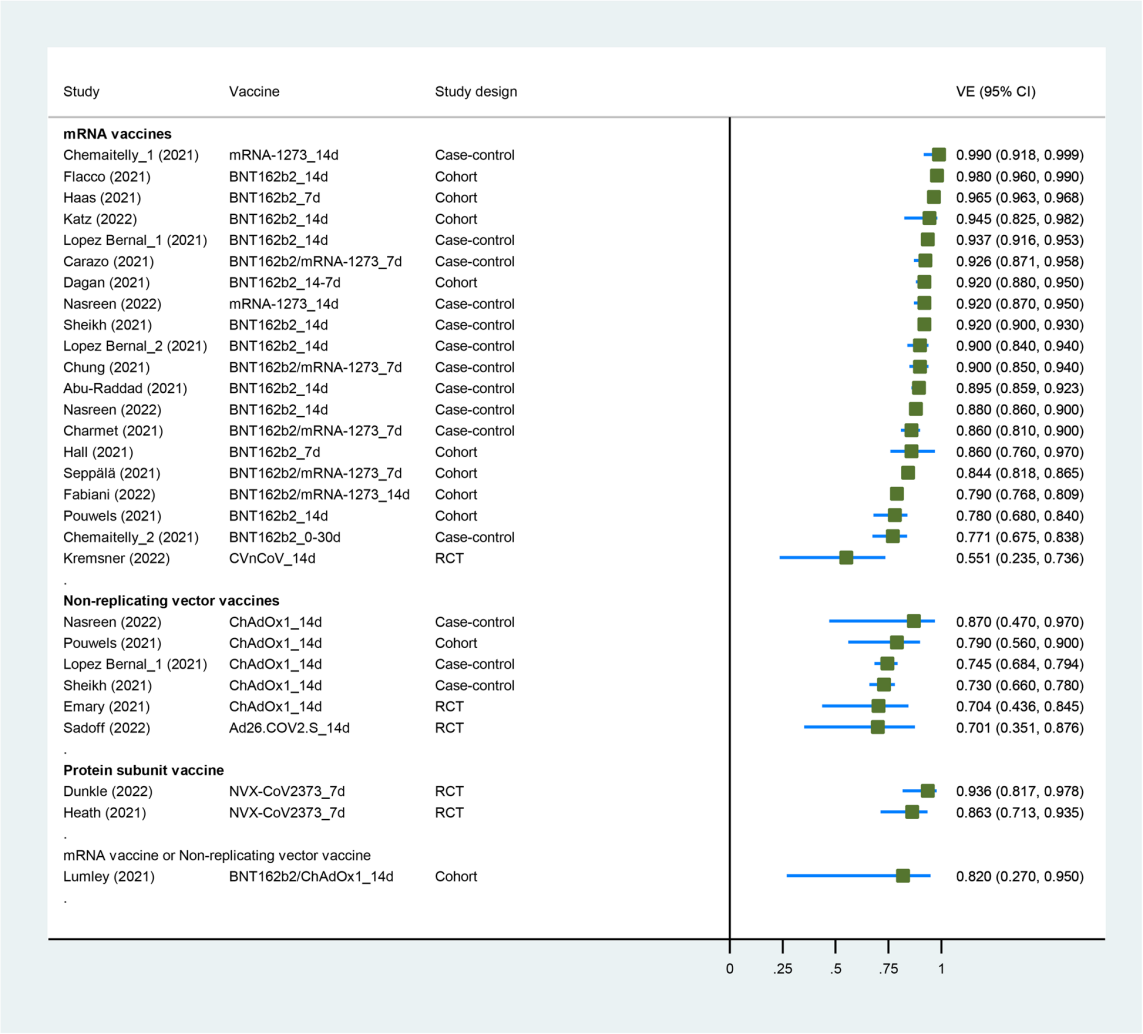
Forest plot showing VE of full vaccination against Alpha variant. Abbreviations: VE, vaccine effectiveness; CI, confidence interval; RCT, randomized controlled trial

Five RCTs [23, 36, 38–40], 9 cohort studies [6, 7, 42, 43, 46, 48, 49, 53, 56], and 10 case-control studies [21, 60, 64–68, 76, 77, 80] had evaluated the VE of COVID-19 vaccines against the Alpha variant. Six COVID-19 vaccines (BNT162b2, mRNA-1273, NVX-CoV2373, ChAdOx1, Ad26.COV2.S and CVnCoV) were included in this analysis. Four studies enrolled healthcare workers [6, 48, 49, 64], one enrolled adults aged 70 or older [76], and the others enrolled the general population. Characteristics of individual studies and VE for Alpha variant are summarized in Fig. 1 and Additional file 1 (Table S4).

The summary VE of full vaccination against the Alpha variant was 88.0% (95% CI, 83.0–91.5) (Table 2). The VE against any infection and symptomatic infection with the Alpha variant was 89.4% (95% CI, 82.9–93.5) and 90.9% (95% CI, 84.5–94.7), respectively. Subgroup analysis by study design showed that VE was 77.9% (59.3–88.0) in 5 RCTs and 89.3% (95% CI, 84.4–92.6) in 19 real-world settings (case-control or cohort studies) (*p*_interaction_ = 0.067). The VE against Alpha variant of real-world evidence seems to be higher than RCTs. Subgroup analysis of vaccine type showed that VE was 90.1% (95% CI, 85.2–93.4) for mRNA vaccines in 20 study groups, 73.9% (95% CI, 69.9–77.4) for non-replicating vector vaccines in 6 study groups, 89.7% (95% CI, 78.8–95.0) for protein subunit vaccine in 2 study groups, and 82.0% (95% CI, 27.0–95.0) for mixed vaccines (BNT162b2/ChAdOx1) in 1 study group (*p*_interaction_ = 0.148). And we detected a significant interaction (*p*_interaction_ = 0.026) between VE and vaccine type (mRNA vaccines vs. not mRNA vaccines); the VE of mRNA vaccines seemed to be higher than others. The results of subgroup analysis for participant are shown in Table 2.

**Table 2:** Meta-analysis and subgroup analysis for VE of COVID-19 against VOC

Covariates	Subgroup	Study groups	Pooled estimates	*I*^2^	*p* (ES=1)	VE% (95% CI)	*P*_interaction_
**Alpha**							
All		29	0.120 (0.085–0.170)	98%	< 0.001	88.0 (83.0–91.5)	
	Any infection	17	0.106 (0.065–0.171)	99%	< 0.001	89.4 (82.9–93.5)	
	Symptomatic	18	0.091 (0.053–0.155)	98%	< 0.001	90.9 (84.5–94.7)	
Study design	RWE	24	0.107 (0.074–0.156)	98%	< 0.001	89.3 (84.4–92.6)	**0.067**
	RCT	5	0.221 (0.120–0.407)	71%	< 0.001	77.9 (59.3–88.0)	
Participant	GP	24	0.125 (0.085–0.182)	98%	< 0.001	87.5 (81.8–91.5)	0.553
	HCWs	4	0.087 (0.056–0.133)	0%	< 0.001	91.3 (86.7–94.4)	
	Older	1	0.100 (0.061–0.163)	–	< 0.001	90.0 (83.7–93.9)	
Vaccine type	mRNA vaccines (BNT162b2/mRNA-1273/CVnCoV)	20	0.099 (0.066–0.148)	98%	< 0.001	90.1 (85.2–93.4)	0.148
	Non-replicating vector vaccine (ChAdOx1/Ad26.COV2.S)	6	0.261 (0.226–0.301)	0%	< 0.001	73.9 (69.9–77.4)	
	Protein subunit vaccine (NVX-CoV2373)	2	0.103 (0.050–0.212)	25%	< 0.001	89.7 (78.8–95.0)	
	Mixed (BNT162b2/ChAdOx1)	1	0.180 (0.047–0.688)	–	0.012	82.0 (31.2–95.3)	
	*Not mRNA vaccines*	9	0.234 (0.190–0.288)	28%	< 0.001	76.6 (71.2–81.0)	**0.026**^**a**^
**Beta**							
All	GP	11	0.270 (0.205–0.357)	82%	< 0.001	73.0 (64.3–79.5)	
	Any infection	7	0.214 (0.169–0.272)	74%	< 0.001	78.6 (72.8–83.1)	**0.044**
	Symptomatic	4	0.608 (0.411–0.899)	20%	0.013	39.2 (10.1–58.9)	
Study design	RWE	7	0.221 (0.178–0.274)	68%	< 0.001	77.9 (72.6–82.2)	**0.032**
	RCT	4	0.544 (0.282–1.051)	65%	0.070	45.6 (-5.1–71.8)	
Vaccine type	mRNA vaccines (BNT162b2/mRNA-1273)	8	0.214 (0.170–0.270)	70%	< 0.001	78.6 (73.0–83.0)	**0.057**
	Non-replicating vector vaccine (ChAdOx1/Ad26.COV2.S)	2	0.690 (0.477–1.000)	0%	0.050	31.0 (0.0–52.3)	
	Protein subunit vaccine (NVX-CoV2373)	1	0.489 (0.238–1.006)	–	0.052	51.1 (-0.6–76.2)	
**Gamma**							
All		10	0.370 (0.263–0.521)	78%	< 0.001	63.0 (47.9–73.7)	
	Any infection	3	0.438 (0.295–0.650)	0%	< 0.001	56.2 (35.0–70.5)	0.960
	Symptomatic	7	0.363 (0.238–0.553)	85%	< 0.001	63.7 (44.7–76.2)	
Study design	RWE	6	0.340 (0.209–0.551)	86%	< 0.001	66.0 (44.9–79.1)	0.634
	RCT	4	0.451 (0.269–0.757)	40%	0.003	54.9 (24.3–73.1)	
Participant	GP	5	0.287 (0.130–0.633)	78%	0.002	71.3 (36.7–87.0)	0.391
	Older/LTCH	4	0.378 (0.228–0.628)	87%	< 0.001	62.2 (37.2–77.2)	
	HCWs	1	0.632 (0.258–1.549)	–	0.316	36.8 (-54.9–74.2)	
Vaccine type	mRNA vaccines (BNT162b2/mRNA-1273/CVnCoV)	4	0.285 (0.147–0.555)	68%	< 0.001	71.5 (44.5–85.3)	0.232
	Non-replicating vector vaccine (ChAdOx1/Ad26.COV2.S)	3	0.373 (0.163–0.852)	91%	0.019	62.7 (14.8–83.7)	
	Inactivated vaccine (CoronaVac)	2	0.534 (0.465–0.614)	0%	< 0.001	46.6 (38.6–53.5)	
	Protein subunit vaccine (SCB-2019)	1	0.082 (0.005–1.361)	–	0.081	91.8 (-36.1–99.5)	
**Beta/Gamma**							
All		21	0.307 (0.238–0.396)	88%	< 0.001	69.3 (60.4–76.2)	
Vaccine type	mRNA vaccines	12	0.228 (0.182–0.287)	69%	< 0.001	77.2 (71.3–81.8)	**0.006**^**a**^
	*Not mRNA vaccines*	9	0.492 (0.360–0.674)	75%	< 0.001	50.8 (32.6–64.0)	
**Delta**							
All		47	0.222 (0.180–0.273)	99%	< 0.001	77.8 (72.7–82.0)	
	Any infection	28	0.254 (0.215–0.300)	95%	< 0.001	74.6 (70.0–78.5)	
	Symptomatic	24	0.208 (0.154–0.281)	99%	< 0.001	79.2 (71.9–84.6)	
Participant	GP	35	0.245 (0.193–0.312)	99%	< 0.001	75.5 (68.8–80.7)	
	HCWs	5	0.236 (0.112–0.497)	93%	< 0.001	76.4 (50.3–88.8)	
	Older	5	0.403 (0.296–0.550)	98%	< 0.001	59.7 (45.0–70.4)	
	Adolescents	7	0.112 (0.076–0.165)	92%	< 0.001	88.8 (83.5–92.4)	
Study design	REW	44	0.214 (0.173–0.266)	99%	< 0.001	78.6 (73.4–82.7)	0.168
	RCT	3	0.407 (0.176–0.940)	70%	< 0.001	59.3 (6.0–82.4)	
Vaccine type	mRNA vaccines (BNT162b2/mRNA-1273)	28	0.166 (0.134–0.204)	99%	< 0.001	83.4 (79.6–86.6)	**< 0.001**
	Non-replicating vector vaccine (ChAdOx1/Ad26.COV2.S)	12	0.350 (0.276–0.445)	98%	< 0.001	65.0 (55.5–72.4)	
	Inactivated vaccine (CoronaVac/HB02/CNBG/BBV152/BBIBP-CorV)	6	0.433 (0.358–0.523)	0%	< 0.001	56.7 (47.7–64.2)	
	Protein subunit vaccine (SCB-2019)	1	0.213 (0.101–0.449)	–	< 0.001	78.7 (55.1–89.9)	
	*Not mRNA vaccines*	19	0.368 (0.303–0.448)	97%	< 0.001	63.2 (55.2–69.7)	**< 0.001**^**a**^
**Delta (booster vaccination)**							
All		6	0.045 (0.035–0.058)	95%	< 0.001	95.5 (94.2–96.5)	
**Omicron**							
All		3	0.441 (0.330–0.591)	90%	< 0.001	55.9 (40.9–67.0)	
**Omicron (booster vaccination)**							
All		2	0.192 (0.089–0.414)	99%	< 0.001	80.8 (58.6–91.1)	

Abbreviations: *VE* vaccine effectiveness, *HCWs* healthcare workers, *LTCH* long-term care homes, *GP* general population, *RCT* randomized controlled trial, *ES* effect size

^a^*P* for interaction between vaccine effectiveness and vaccine type (mRNA vaccines vs. not mRNA vaccines)

### Vaccine effectiveness of COVID-19 vaccines against B.1.351 (Beta) and P.1 (Gamma) variants

**Fig. 2:**
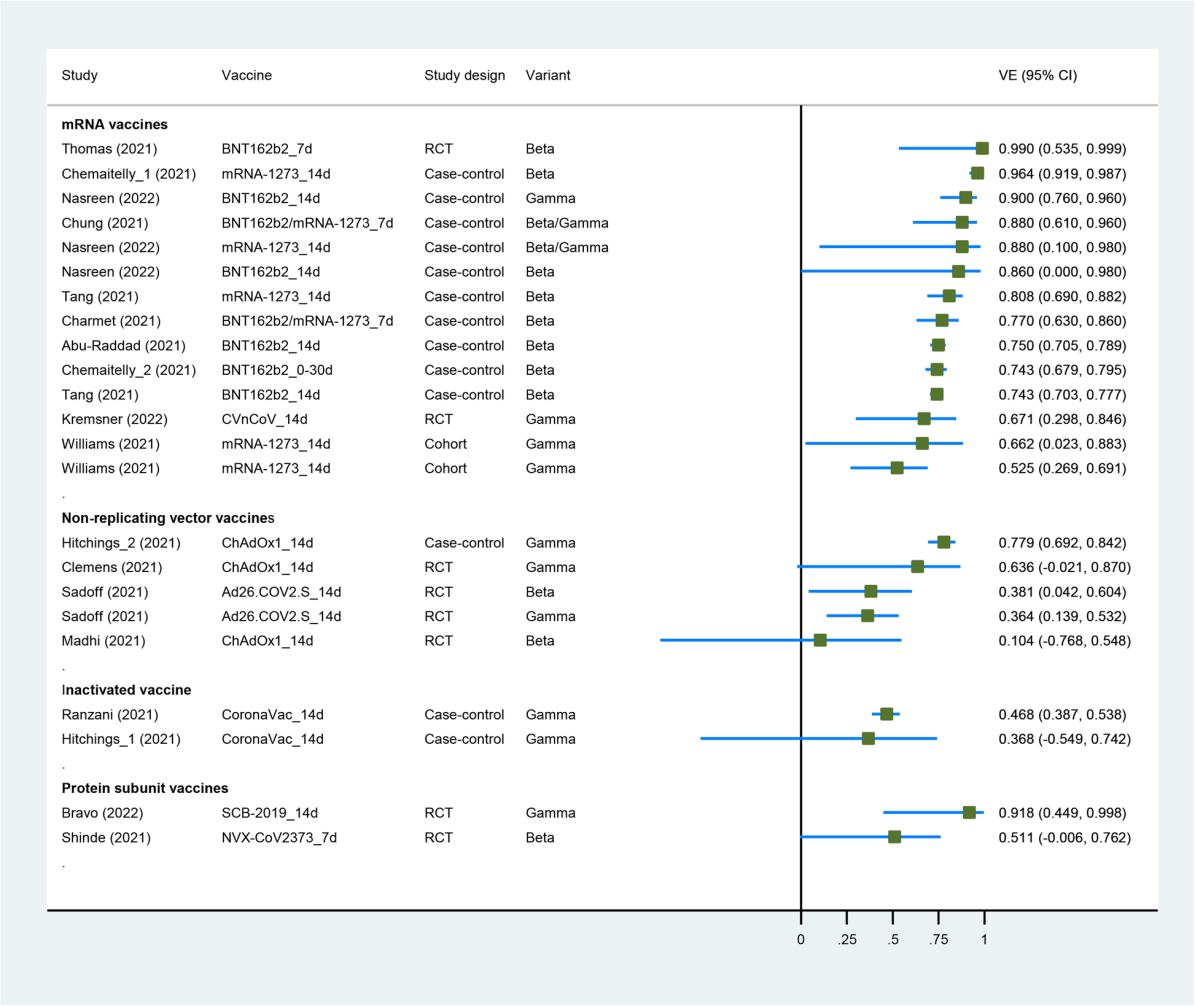
Forest plot showing VE of full vaccination against Beta/Gamma variants. Abbreviations: VE, vaccine effectiveness; CI, confidence interval; RCT, randomized controlled trial

Four RCTs [20, 22, 40, 41] and 6 case-control studies [60, 65–67, 77, 82] had evaluated the VE of COVID-19 vaccines against the Beta variant. Four RCTs [34, 35, 39, 40], 1 cohort study [58], and 4 case-control studies [72, 73, 77, 79] had evaluated the VE of COVID-19 vaccines against the Gamma variant. Both Beta and Gamma have N501Y and E484K mutations, and 2 studies used a combined Beta/Gamma group because of insufficient specimens. Eight COVID-19 vaccines (mRNA-1273, BNT162b2, NVX-CoV2373, ChAdOx1, CVnCoV, SCB-2019, CoronaVac, and Ad26.COV2.S) were included in this analysis. For the study population, 1 study enrolled health care workers [73], 2 studies enrolled older adults [72, 79], 1 studies enrolled participants from long-term care homes [58], and the others enrolled the general population. Characteristics of individual studies and VE for Beta and Gamma variants are summarized in Fig. 2 and Additional file 1 (Table S5).

The summary VE of full vaccination against Beta variant was 73.0% (95% CI, 64.3–79.5) (Table 2). Subgroup analysis by study design showed that VE for Beta variant was 45.6% (95% CI, −5.1 to 71.8) in 4 RCTs and 77.9% (95% CI, 72.6–82.2) in 7 real-world settings (case-control studies) (*p*_interaction_ = 0.067). The VE against Beta variant of real-world evidence seems to be higher than RCTs. Subgroup analysis of vaccine type showed that VE was 78.6% (95% CI, 73.0–83.0) for mRNA vaccines in 8 study groups, 31.0% (95% CI, 0.0–52.3) for non-replicating vector vaccines in 2 study groups, and 51.1% (95% CI, −0.6 to 76.2) for protein subunit vaccine in 1 study group (*p*_interaction_ = 0.057). The summary VE of full vaccination against Gamma variant was 63.0% (95% CI, 47.9–73.7) in 10 study groups; the results of subgroup analysis are shown in Table 2.

When Beta/Gamma variant was treated as one group, the summary VE of full vaccination was 69.3% (95% CI, 60.4–76.2) in 21 study groups. Subgroup analysis of vaccine types showed that VE against Beta/Gamma was 77.2% (95% CI, 71.3–81.8) for mRNA vaccines in 12 study groups and 50.8% (95% CI, 32.6–64.0) for not mRNA vaccines in 9 study groups (*p*_interaction_ = 0.006); the VE for mRNA vaccines seemed to be higher than others.

### Vaccine effectiveness of COVID-19 vaccines against B.1.617.2 (Delta) variant

**Fig. 3:**
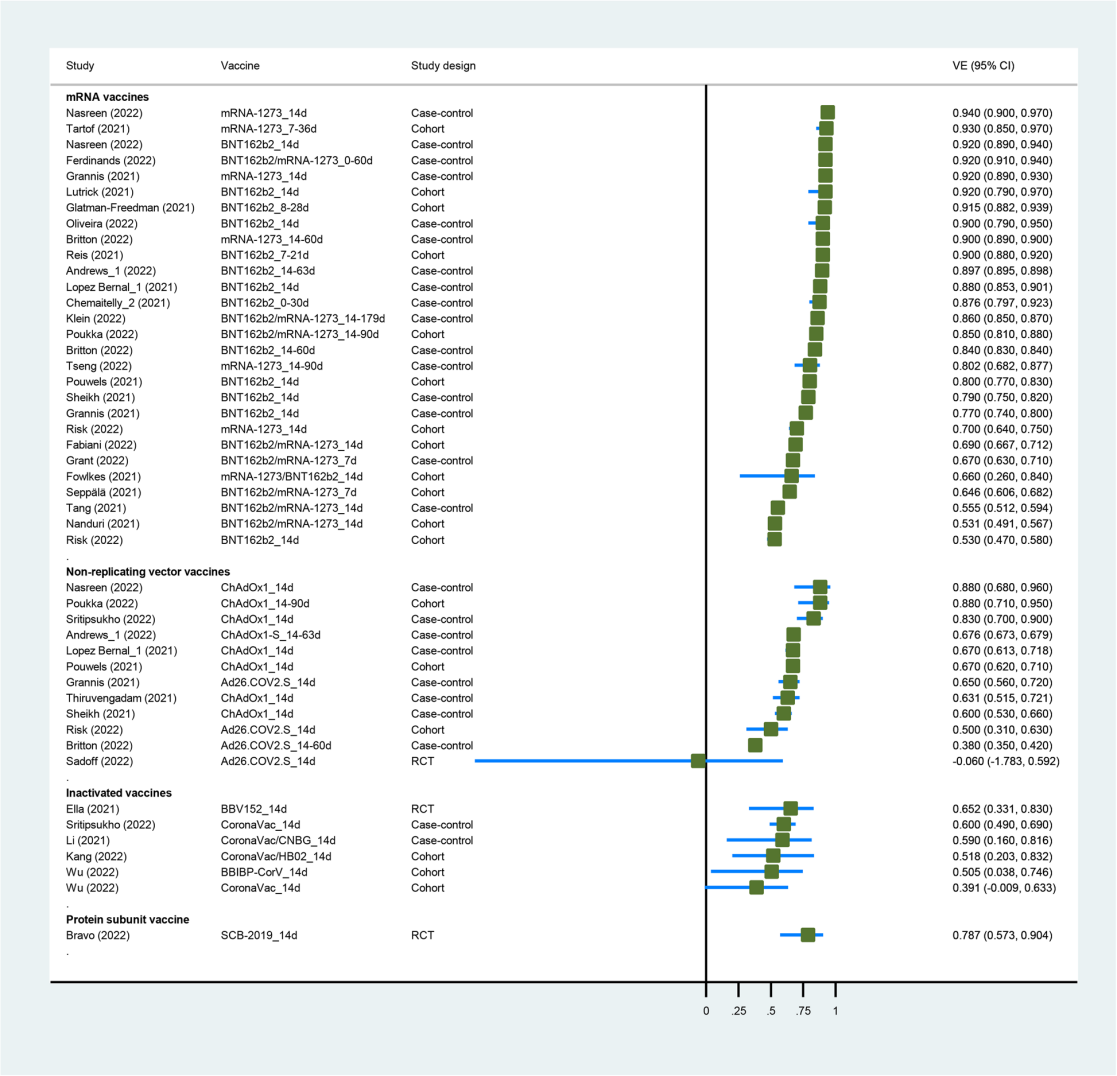
Forest plot showing VE of full vaccination against Delta variant. Abbreviations: VE, vaccine effectiveness; CI, confidence interval; RCT, randomized controlled trial

**Fig. 4:**
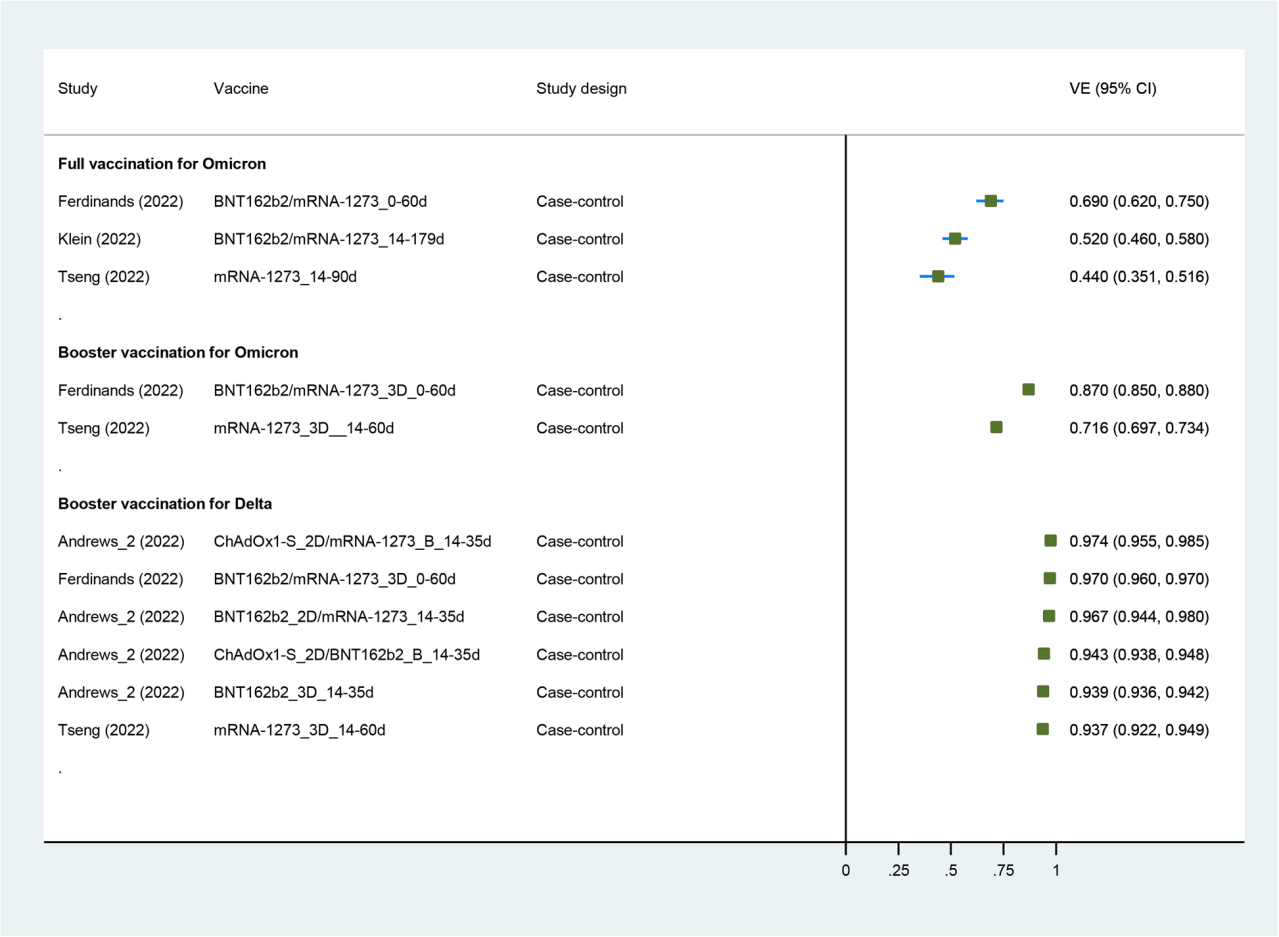
Forest plot showing VE of full vaccination against Omicron variant, and VE of booster vaccination against Delta or Omicron variant. Abbreviations: VE, vaccine effectiveness; CI, confidence interval; RCT, randomized controlled trial

Three RCTs [34, 37, 40], 13 cohort studies [42, 44, 45, 47, 50–57, 59], and 17 case-control studies [21, 61–63, 66, 69–71, 74, 75, 77, 78, 80–84] had evaluated the VE of COVID-19 vaccines against the Delta variant. Ten COVID-19 vaccines (mRNA-1273, BBV152, ChAdOx1, BNT162b2, CoronaVac, Ad26.COV2.S, HB02, CNBG, SCB-2019, and BBIBP-CorV) were included in this analysis. Five studies enrolled adolescents [45, 50, 54, 63, 78], 3 studies enrolled health care workers or frontline workers [42, 44, 52], 1 study enrolled participants in the nursing home [51], and the others enrolled the general population. Three studies reported the VE of booster vaccination against Delta variant [61, 69, 84]. Characteristics of individual studies and VE for Delta variant are summarized in Figs. 3 and 4 and Additional file 1 (Table S6).

The summary VE of full vaccination against the Delta variant was 77.8% (95% CI, 72.7–82.0) (Table 2). The VE against any infection and symptomatic infection with the Delta variant were 74.6% (95% CI, 70.0–78.5) and 79.2% (95% CI, 71.9–84.6), respectively. For special populations, the VE was 76.4% (95% CI, 50.3–88.8) in healthcare workers, 59.7% (95% CI, 45.0–70.4) in older adults, and 88.8% (95% CI, 83.5–92.4) in adolescents. Subgroup analysis of study design showed that VE for Delta variant was 59.3% (95% CI, 6.0–82.4) in 3 RCTs and 78.6% (95% CI, 73.4–82.7) in 30 real-world settings (case-control or cohort studies) (*p*_interaction_ = 0.168). Subgroup analysis by vaccine type showed that VE was 83.4% (95% CI, 79.6–86.6) for mRNA vaccines in 28 study groups, 65.0% (95% CI, 55.5–72.4) for non-replicating vector vaccines in 12 study groups, 56.7% (95% CI, 47.7–64.2) for inactivated vaccines in 6 study groups, and 78.7% (95% CI, 55.1–89.9) for protein subunit vaccine in 1 study group (*p*_interaction_ < 0.001). An interaction (*p*_interaction_ < 0.001) between VE and vaccine type (mRNA vaccines vs. not mRNA vaccines) was found; the VE for mRNA vaccines seemed to be higher than others. The summary VE of booster vaccination against the Delta variant was 95.5% (95% CI, 94.2–96.5).

### Vaccine effectiveness of COVID-19 vaccines against B.1.1.529 (Omicron) variant

Three case-control studies had evaluated the VE of COVID-19 vaccines against the Omicron variant [69, 74, 84]. Two mRNA vaccines (mRNA-1273 and BNT162b2) were included in this analysis. All three studies enrolled the general population. Characteristics of individual studies and VE for Omicron variant are summarized in Fig. 4 and Additional file 1 (Table S7). The summary VE of full vaccination against the Omicron variant was 55.9% (95% CI, 40.9–67.0), and the VE of booster vaccination against the Omicron variant was 80.8% (95% CI, 58.6–91.1).

## Discussion

The VOC have mutations in its spike protein; most breakthrough cases were caused by contemporary variant strains [36]. The VE of current COVID-19 vaccines against VOC is concerning; we conducted this systematic review and meta-analysis to synthesize evidence on this topic during the pandemic. This study has five main findings. First, full vaccination of COVID-19 vaccines was effective against Alpha, Beta, Gamma, Delta, and Omicron variants, with the VE of 88.0%, 73.0%, 63.0%, 77.8%, and 55.9%, respectively. Second, booster vaccination has higher VE against Delta and Omicron variants, with the VE of 95.5% and 80.8%, respectively. Third, mRNA vaccines (BNT162b2 or mRNA-1273) have higher VE against VOC over other vaccines. Fourth, VE against VOC of real-world evidence seemed to be higher than RCTs. Fifth, more evidence was needed to evaluate the VE of COVID-19 against the Omicron variant. To our knowledge, our study is the first comprehensive systematic review and meta-analysis to characterize the VE of COVID-19 vaccines against five kinds of VOC.

The evidence for the Omicron variant was insufficient, which only included three studies evaluating the VE of the mRNA vaccines (BNT162b2 or mRNA-1273). WHO guidelines recommend a lower bound of at least 30% and a vaccine efficacy of at least 50% [85]. The summary VE against Omicron variant was 55.9% of full vaccination and 80.8% of booster vaccination, raising concern for other vaccines. One study showed that Omicron variant extensively but incompletely escaped BNT162b2 neutralization [86]. Owing to multiple spike mutations, over 85% of tested neutralizing antibodies were escaped by Omicron variant, presenting a serious threat to existing therapies and COVID-19 vaccines [12, 87].

The main results in this study were in consistent with a recent meta-analysis for neutralizing antibodies against SARS-CoV-2 variants, which showed that Alpha, Beta, Gamma, and Delta variants significantly escaped natural-infection-mediated neutralization, with an average of 1.4-fold, 4.1-fold, 1.8-fold, and 3.2-fold reduction in live virus neutralization assay [88]. Despite the reduction in neutralization titers against Alpha variant, they remain robust, and there is no evidence of vaccine escape in one study [89]. Escape of Beta variant from neutralization by convalescent plasma and vaccine-induced sera was observed in some studies [13, 90, 91]. Although neutralization titers against Gamma variant are reduced, it is hoped that immunization with vaccines designed against parent strains will protect Gamma variant infection [92]. The Delta variant escapes neutralization by some antibodies that target the receptor-binding domain or N-terminal domain; the neutralization titers against Delta were three to fivefolds than Alpha variant when two-dose of the vaccine administrated [15]. This study also supports the two-dose vaccine regimen recommended by the FDA and EMA, which is consistent with an in vitro study for SARS-CoV-2 variants of concern [93]. Also, booster vaccination demonstrates high VE against Delta infection in our study.

We did not evaluate VE against asymptomatic infection due to poor reporting in included studies. The summary VE against asymptomatic infection was slightly higher than any infection for Alpha, Gamma, and Delta variants, which was consistent with primary studies. The summary VE was higher against any infection with the Beta variant, which was probably confounded by study design and vaccine type. Three of 4 RCTs used symptomatic infection as an outcome, but 5 of 6 case-control studies used any infection. Most studies enrolled general population; only a few studies analyzed the VE in older adults or adolescents. We have performed subgroup analyses for VE against Delta variant stratifying by participants; the VE was 59.7% in older adults which was lower than general population (75.5%), healthcare workers (76.4%), and adolescents (88.8%). Vaccine type may be a confounder for this analysis, because one study showed that the VE of Ad26.COV2.S was much lower than BNT162b2 and mRNA-1273 in adolescents [63]. More evidence is needed for evaluating the VE against VOC in special population.

This review included 3 study designs evaluating 11 COVID-19 vaccines against 5 VOC in different populations. There is high heterogeneity between studies, and high statistical heterogeneity is also observed in most analysis. Other factors like the definition of outcomes (all SARS-CoV-2 or symptomatic infection), days after vaccination, and participant’s characteristics (e.g., age and race) may also contribute to the heterogeneity. Therefore, we mainly performed narrative descriptive synthesis.

This study has some limitations. First, 19% of studies (11 of 57) are nonrandomized. The imbalance between groups in observational studies is a concern, so potential selection bias may be existent. Second, we did not evaluate VE against asymptomatic infection due to poor reporting in included studies. Third, although we performed qualitative analysis by different stratifications, heterogeneity was still high in most quantitative analysis. Fourth, VE against hospitalization or death related to VOC is not included in our analysis. Finally, the evidence of COVID-19 vaccines against Omicron variant is not enough, more research is needed in the future.

## Conclusions

Full vaccination of COVID-19 vaccines is highly effective against Alpha variant and moderate effective against Beta, Gamma, and Delta variant. Booster vaccination has more effectiveness against Delta and Omicron variants. mRNA vaccines (BNT162b2 or mRNA-1273) seem to have higher VE against Alpha, Beta, Gamma, or Delta over other vaccines. SARS-CoV-2 Omicron is raising concern for vaccinated individuals, and more evidence is needed to evaluate the VE of COVID-19 vaccines against the Omicron variant.

## Supplementary Information

**Additional file 1: Supplementary Materials.** Search strategy (**Appendix S1**). Flow chart of literature search and study selection (**Figure S1**). Risk of bias for included randomized controlled trials (**Table S1**). Risk of bias for included cohort studies (**Table S2**). Risk of bias for included case-control studies (**Table S3**). VE of COVID-19 vaccines against Alpha variant (**Table S4**). VE of COVID-19 vaccines against Beta and Gamma variant (**Table S5**). VE of COVID-19 vaccines against Delta variant (**Table S6**). VE of COVID-19 vaccines against Omicron variant (**Table S7**).

## Data Availability

The datasets used and/or analyzed during the current study are available from the corresponding author on reasonable request.

## Abbreviations

CI: Confidence interval
NOS: Newcastle-Ottawa scale
PRISMA: Preferred Reporting Items for Systematic reviews and Meta-Analyses
RCT: Randomized controlled trial
VE: Vaccine effectiveness
VOC: Variants of concern
