# Supplementary material for: Effectiveness of COVID-19 vaccines against SARS-CoV-2 variants of concern: a systematic review and meta-analysis
Source: BMC Med. 2022 May 23;20:200. doi: 10.1186/s12916-022-02397-y (PMC9126103; doi:10.1186/s12916-022-02397-y)
Supplement: Supplementary file 1 — Additional file 1: Supplementary Materials. Search strategy (Appendix S1). Flow chart of literature search and study selection (Figure S1). Risk of bias for included randomized controlled trials (Table S1). Risk of bias for included cohort studies (Table S2). Risk of bias for included case-control studies (Table S3). VE of COVID-19 vaccines against Alpha variant (Table S4). VE of COVID-19 vaccines against Beta and Gamma variant (Table S5). VE of COVID-19 vaccines against Delta variant (Table S6). VE of COVID-19 vaccines against Omicron variant (Table S7). [file 12916_2022_2397_MOESM1_ESM.doc]

## Supplementary Material

### Appendix S1. Search strategy

**Search strategy for Embase (3318 items)**

1. 'coronavirus disease 2019'/exp OR 'Severe acute respiratory syndrome coronavirus 2'/exp OR (COVID-19 OR SARS-CoV-2):ti,ab

2. vaccine/exp OR vaccination/exp OR immunization/exp OR (vaccin* OR immuniz*):ti,ab

3. (variant* OR B.1.1.7 OR B.1.351 OR P.1 OR B.1.617.2 OR B.1.1.529):ti,ab

4. #1 AND #2 AND #3

**Search strategy for PubMed (3231 items)**

1. COVID-19[Mesh] OR SARS-CoV-2[Mesh] OR "COVID-19 Vaccines"[Mesh] OR COVID-19[Title/Abstract] OR SARS-CoV-2[Title/Abstract]

2. vaccines[Mesh] OR vaccination[Mesh] OR immunization[Mesh] OR vaccin*[Title/Abstract] OR immuniz*[Title/Abstract]

3. variant*[Title/Abstract] OR B.1.1.7[Title/Abstract] OR B.1.351[Title/Abstract] OR P.1[Title/Abstract] OR B.1.617.2[Title/Abstract] OR B.1.1.529[Title/Abstract]

4. #1 AND #2 AND #3

**Search strategy for Cochrane Library (71 items)**

1. [mh COVID-19]

2. [mh SARS-CoV-2]

3. COVID-19:ti,ab

4. SARS-CoV-2:ti,ab

5. #1 OR #2 OR #3 OR #4

6. [mh vaccines]

7. [mh vaccination]

8. [mh immunization]

9. vaccin*:ti,ab

10. immuniz*:ti,ab

11. #6 OR #7 OR #8 OR #9 OR #10

12. (variant* OR B.1.1.7 OR B.1.351 OR P.1 OR B.1.617.2 OR B.1.1.529):ti,ab

13. #5 AND #11 AND #12

**Search strategy for Clinicaltrial.gov (120 items)**

(vaccines OR vaccine OR vaccination OR immunization) AND (variant OR variants) AND (COVID-19 OR SARS-CoV-2)

### Supplementary Figure


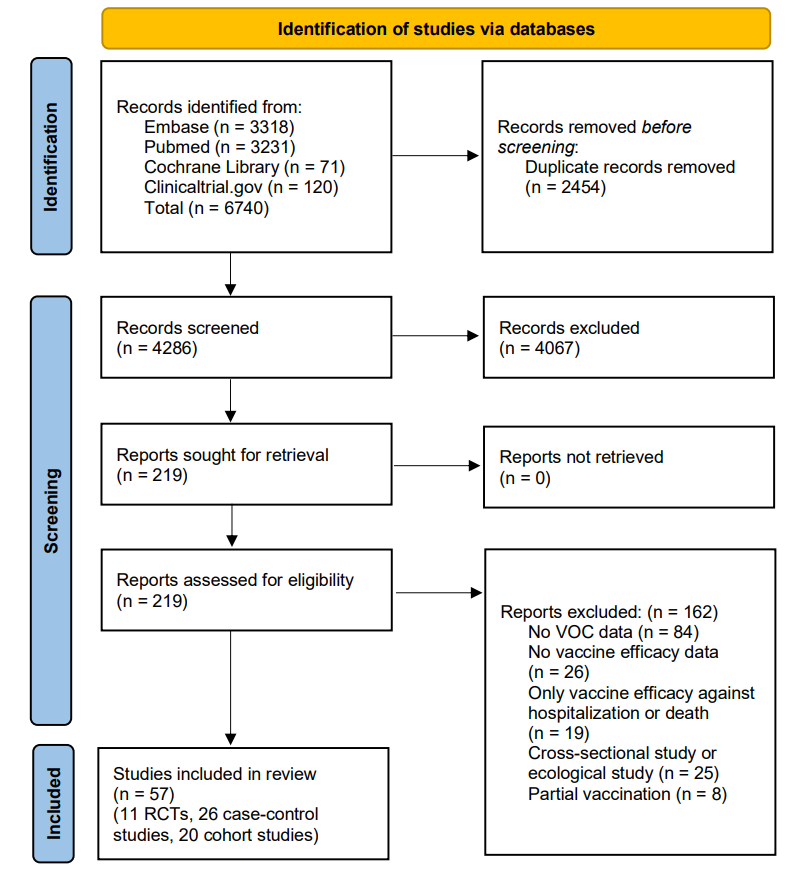


Figure S1. Flow chart of literature search and study selection.

### Supplementary Tables

**Table S1**. Risk of bias for included RCTs

| Study (First author) | Randomization process | Deviations from intended interventions | Missing outcome data | Measurement of the outcome | Selection of the reported result | **Overall bias** |
| --- | --- | --- | --- | --- | --- | --- |
| Heath (2021) | Low | Some concerns | Some concerns | Low | Some concerns | Some concerns |
| Emary (2021) | Low | Some concerns | Some concerns | Low | Some concerns | Some concerns |
| Shinde (2021) | Low | Low | Some concerns | Low | Some concerns | Some concerns |
| Madhi (2021) | Low | Some concerns | Low | Low | Some concerns | Some concerns |
| Sadoff (2022) | Low | Some concerns | Some concerns | Low | Low | Some concerns |
| Thomas (2021) | Low | Some concerns | Low | Low | Low | Some concerns |
| Ella (2021) | Low | Some concerns | Some concerns | Low | Low | Some concerns |
| Clemens (2021) | Low | Some concerns | Low | Low | Some concerns | Some concerns |
| Bravo (2022) | Low | Some concerns | Low | Low | Low | Some concerns |
| Dunkle (2022) | Low | Some concerns | Low | Low | Low | Some concerns |
| Kremsner (2022) | Low | Some concerns | Low | Low | Low | Some concerns |

**Table S2**. Risk of bias for included cohort studies

|  | Selection | | | | Comparability | Exposure | | |  |  |
| --- | --- | --- | --- | --- | --- | --- | --- | --- | --- | --- |
|  | Item 1 | Item 2 | Item 3 | Item 4 | Item 5 | Item 6 | Item 7 | Item 7 | Total score | **Quality** |
| Hall (2021) | 1 | 1 | 1 | 1 | 1 | 1 | 1 | 1 | 8 | Good |
| Haas (2021) | 1 | 1 | 1 | 0 | 1 | 1 | 1 | 1 | 7 | Good |
| Lumley (2021) | 1 | 1 | 1 | 0 | 1 | 1 | 1 | 1 | 7 | Good |
| Williams (2021) | 0 | 1 | 1 | 0 | 0 | 0 | 1 | 1 | 4 | Moderate |
| Nanduri (2021) | 1 | 1 | 1 | 0 | 1 | 1 | 1 | 1 | 7 | Good |
| Fowlkes (2021) | 0 | 1 | 1 | 0 | 1 | 0 | 1 | 1 | 5 | Moderate |
| Dagan (2021) | 1 | 1 | 1 | 0 | 2 | 1 | 1 | 1 | 8 | Good |
| Pouwels (2021) | 1 | 1 | 1 | 0 | 2 | 1 | 1 | 1 | 8 | Good |
| Flacco (2021) | 1 | 1 | 0 | 0 | 0 | 0 | 1 | 1 | 4 | Moderate |
| Glatman-Freedman (2021) | 1 | 1 | 1 | 0 | 1 | 1 | 1 | 1 | 7 | Good |
| Seppälä (2021) | 1 | 1 | 1 | 0 | 1 | 1 | 1 | 1 | 7 | Good |
| Fabiani (2022) | 1 | 1 | 1 | 0 | 2 | 1 | 1 | 1 | 8 | Good |
| Risk (2022) | 1 | 1 | 1 | 0 | 2 | 1 | 1 | 1 | 8 | Good |
| Kang (2022) | 1 | 1 | 1 | 0 | 1 | 1 | 1 | 1 | 7 | Good |
| Poukka (2022) | 1 | 1 | 1 | 0 | 1 | 1 | 1 | 1 | 7 | Good |
| Wu (2022) | 0 | 1 | 1 | 0 | 1 | 1 | 1 | 1 | 6 | Moderate |
| Katz (2022) | 0 | 1 | 1 | 1 | 1 | 0 | 1 | 1 | 6 | Moderate |
| Lutrick (2021) | 0 | 1 | 1 | 1 | 1 | 1 | 1 | 1 | 7 | Good |
| Reis (2021) | 1 | 1 | 1 | 0 | 1 | 1 | 1 | 1 | 7 | Good |
| Tartof (2021) | 1 | 1 | 1 | 0 | 2 | 1 | 1 | 1 | 8 | Good |

Item 1: Representativeness of the exposed cohort.

Item 2: Selection of the non-exposed cohort.

Item 3: Ascertainment of exposure.

Item 4: Demonstration that outcome of interest was not present at start of study.

Item 5: Comparability of cohorts on the basis of the design or analysis.

Item 6: Assessment of outcome.

Item 7: Was follow up long enough for outcomes to occur.

Item 8: Adequacy of follow up of cohorts.

**Table S3**. Risk of bias for included case-control studies

|  | Selection | | | | Comparability | Exposure | | |  |  |
| --- | --- | --- | --- | --- | --- | --- | --- | --- | --- | --- |
|  | Item 1 | Item 2 | Item 3 | Item 4 | Item 5 | Item 6 | Item 7 | Item 7 | Total score | **Quality** |
| Charmet (2021) | 1 | 1 | 1 | 0 | 1 | 1 | 0 | 1 | 6 | Moderate |
| Nasreen (2022) | 1 | 1 | 1 | 1 | 1 | 1 | 1 | 1 | 8 | Good |
| Hitchings_1 (2021) | 1 | 0 | 1 | 1 | 0 | 1 | 1 | 1 | 6 | Moderate |
| Ranzani (2021) | 1 | 0 | 1 | 1 | 2 | 1 | 1 | 1 | 8 | Good |
| Lopez Bernal_1 (2021) | 1 | 1 | 1 | 1 | 2 | 1 | 1 | 1 | 9 | Good |
| Tang (2021) | 1 | 1 | 1 | 1 | 2 | 1 | 1 | 1 | 9 | Good |
| Li (2021) | 1 | 0 | 1 | 1 | 1 | 1 | 1 | 1 | 7 | Good |
| Abu-Raddad (2021) | 1 | 1 | 1 | 1 | 2 | 1 | 1 | 1 | 9 | Good |
| Sheikh (2021) | 1 | 0 | 1 | 1 | 0 | 1 | 1 | 1 | 6 | Moderate |
| Chemaitelly_1 (2021) | 1 | 1 | 1 | 1 | 2 | 1 | 1 | 1 | 9 | Good |
| Lopez Bernal_2 (2021) | 1 | 0 | 1 | 1 | 1 | 1 | 1 | 1 | 7 | Good |
| Chung (2021) | 1 | 1 | 1 | 1 | 1 | 1 | 1 | 1 | 8 | Good |
| Carazo (2021) | 1 | 0 | 1 | 1 | 1 | 1 | 1 | 1 | 7 | Good |
| Chemaitelly_2 (2021) | 1 | 1 | 1 | 1 | 2 | 1 | 1 | 1 | 9 | Good |
| Grannis (2021) | 1 | 1 | 1 | 1 | 1 | 1 | 1 | 1 | 8 | Good |
| Sritipsukho (2022) | 1 | 1 | 1 | 1 | 1 | 1 | 1 | 1 | 8 | Good |
| Klein (2022) | 1 | 1 | 1 | 1 | 1 | 1 | 1 | 1 | 8 | Good |
| Oliveira (2022) | 1 | 1 | 1 | 1 | 2 | 1 | 1 | 1 | 9 | Good |
| Tseng (2022) | 1 | 1 | 1 | 1 | 2 | 1 | 1 | 1 | 9 | Good |
| Ferdinands (2022) | 1 | 1 | 1 | 1 | 1 | 1 | 1 | 1 | 8 | Good |
| Britton (2022) | 1 | 1 | 1 | 1 | 2 | 1 | 1 | 1 | 9 | Good |
| Grant (2022) | 1 | 1 | 1 | 0 | 1 | 1 | 0 | 1 | 6 | Moderate |
| Andrews_1 (2022) | 1 | 1 | 1 | 1 | 2 | 1 | 1 | 1 | 9 | Good |
| Andrews_2 (2022) | 1 | 1 | 1 | 1 | 2 | 1 | 1 | 1 | 9 | Good |
| Thiruvengadam (2021) | 1 | 1 | 1 | 1 | 2 | 1 | 1 | 1 | 9 | Good |
| Hitchings_2 (2021) | 1 | 1 | 1 | 1 | 2 | 1 | 1 | 1 | 9 | Good |

Item 1: Is the case definition adequate?

Item 2: Representativeness of the cases.

Item 3: Selection of controls.

Item 4: Definition of controls.

Item 5: Comparability of cases and controls on the basis of the design or analysis.

Item 6: Ascertainment of exposure.

Item 7: Same method of ascertainment for cases and controls.

Item 8: Non-Response Rate.

**Table S4. VE of COVID-19 vaccines against B.1.1.7 (Alpha) variant**

| First author | Study design | Participants, N | Participants | Age  (years) | Vaccine | Outcomes | Day_F | VE% (95% CI) | Variant |
| --- | --- | --- | --- | --- | --- | --- | --- | --- | --- |
| Heath (2021) | RCT | 14,039 | GP | 18-84 | NVX-CoV2373 | Symptomatic | 7d | 86.3 (71.3–93.5) | Alpha |
| Emary (2021) | RCT | 8,534 | GP | ≥18 | ChAdOx1 | Symptomatic | 14d | 70.4 (43.6–84.5) | Alpha |
| Sadoff (2022) | RCT | 4,969 | GP | ≥18 | Ad26.COV2.S | Symptomatic | 14d | 70.1 (35.1–87.6) | Alpha |
| Dunkle (2022) | RCT | 29,949 | GP | ≥18 | NVX-CoV2373 | Symptomatic | 7d | 93.6 (81.7–97.8) | Alpha |
| Kremsner (2022) | RCT | 39,680 | GP | ≥18 | CVnCoV | Symptomatic | 14d | 55.1 (23.5–73.6) | Alpha |
| Hall (2021) | Cohort | 23,324 | HCWs | ≥18 | BNT162b2 | All infections | 7d | 86.0 (76.0–97.0) | Alpha (>50%) |
| Haas (2021) | Cohort | 6,538,911 | GP | ≥16 | BNT162b2 | All infections | 7d | 96.5 (96.3–96.8) | Alpha (95%) |
|  |  |  | GP | ≥16 | BNT162b2 | Symptomatic | 7d | 97.7 (97.5–97.9) | Alpha |
|  |  |  | GP | ≥16 | BNT162b2 | Asymptomatic | 7d | 93.8 (93.3–94.2) | Alpha (51%) |
| Lumley (2021) | Cohort | 13,109 | HCWs | 39 (30-50) ^a^ | BNT162b2/ChAdOx1 | All infections | 14d | 82.0 (27.0–95.0) | Alpha |
| Pouwels (2021) | Cohort | 743,526 | GP | 18-64 | BNT162b2 | All infections | 14d | 78.0 (68.0–84.0) | Alpha (>50%) |
|  |  |  | GP | 18-64 | BNT162b2 | Symptomatic | 14d | 97.0 (96.0–98.0) | Alpha (>50%) |
|  |  |  | GP | 18-64 | ChAdOx1 | All infections | 14d | 79.0 (56.0–90.0) | Alpha (>50%) |
|  |  |  | GP | 18-64 | ChAdOx1 | Symptomatic | 14d | 97.0 (93.0–98.0) | Alpha (>50%) |
| Flacco (2021) | Cohort | 204,840 | GP | ≥18 | BNT162b2 | All infections | 14d | 98.0 (96.0–99.0) | Alpha (83%) |
|  |  |  | GP | ≥18 | BNT162b2 | Symptomatic | 14d | 99.0 (96.0–100.0) | Alpha (83%) |
| Seppälä (2021) | Cohort | 18,431 | GP | ≥18 | BNT162b2/mRNA-1273 | All infections | 7d | 84.4 (81.8–86.5) | Alpha |
| Fabiani (2022) | Cohort | 33,250,344 | GP | ≥16 | BNT162b2/mRNA-1273 | All infections | 14d | 79.0 (76.8–80.9) | Alpha (>50%) |
|  |  |  | Older | 60-79 | BNT162b2/mRNA-1273 | All infections | 14d | 82.2 (80.1–84.1) | Alpha (>50%) |
|  |  |  | Older | ≥80 | BNT162b2/mRNA-1273 | All infections | 14d | 79.8 (76.9–82.4) | Alpha (>50%) |
|  |  |  | HCWs | ≥18 | BNT162b2/mRNA-1273 | All infections | 14d | 82.9 (80.7–84.7) | Alpha (>50%) |
| Katz (2022) | Cohort | 1,250 | HCWs | 45 (36–55) ^a^ | BNT162b2 | All infections | 14d | 94.5 (82.5–98.2) | Alpha (71%) |
|  |  |  | HCWs | 45 (36–55) ^a^ | BNT162b2 | Symptomatic | 14d | 97.0 (72.0–99.7) | Alpha (71%) |
| Dagan (2021) | Cohort | 1,193,236 | GP | ≥16 | BNT162b2 | All infections | 7d | 92.0 (88.0–95.0) | Alpha (80%) |
| Lopez Bernal_1 (2021) | Case-control | 19,109 ^b^ | GP | ≥16 | BNT162b2/ChAdOx1 | Symptomatic | 14d | 87.5 (85.1–89.5) | Alpha |
|  |  |  | GP | ≥16 | BNT162b2 | Symptomatic | 14d | 93.7 (91.6–95.3) | Alpha |
|  |  |  | GP | ≥16 | ChAdOx1 | Symptomatic | 14d | 74.5 (68.4–79.4) | Alpha |
| Abu-Raddad (2021) | Case-control | 35,979 ^b^ | GP | 33 (22-40) ^a^ | BNT162b2 | All infections | 14d | 89.5 (85.9–92.3) | Alpha |
| Lopez Bernal_2 (2021) | Case-control | 3,034 ^b^ | Older | ≥70 | BNT162b2 | Symptomatic | 14d | 90.0 (84.0–94.0) | Alpha |
| Chemaitelly_1 (2021) | Case-control | 66,042 ^b^ | GP | 32 (25-39) ^a^ | mRNA-1273 | All infections | 14d | 100.0 (91.8–100.0) | Alpha |
| Chemaitelly_2 (2021) | Case-control | 113,830 ^b^ | GP | 31 (21-39) ^a^ | BNT162b2 | All infections | 0-30d | 77.1 (67.5–83.8) | Alpha |
| Charmet (2021) | Case-control | 33,863 ^b^ | GP | ≥20 | BNT162b2/mRNA-1273 | All infections | 7d | 86.0 (81.0–90.0) | Alpha |
| Sheikh (2021) | Case-control | 19,543 ^b^ | GP | ≥16 | BNT162b2 | All infections | 14d | 92.0 (90.0–93.0) | Alpha |
|  |  |  | GP | ≥16 | ChAdOx1 | All infections | 14d | 73.0 (66.0–78.0) | Alpha |
| Carazo (2021) | Case-control | 901 ^b^ | HCWs | 18-74 | BNT162b2/mRNA-1273 | All infections | 7d | 92.6 (87.1–95.8) | Alpha |
| Chung (2021) | Case-control | 324,033 ^b^ | GP | ≥16 | BNT162b2/mRNA-1273 | Symptomatic | 7d | 90.0 (85.0–94.0) | Alpha |
| Nasreen (2022) | Case-control | 51,440 ^b^ | GP | ≥16 | BNT162b2 | Symptomatic | 14d | 88.0 (86.0–90.0) | Alpha |
|  |  |  | GP | ≥16 | mRNA-1273 | Symptomatic | 14d | 92.0 (87.0–95.0) | Alpha |
|  |  |  | GP | ≥16 | ChAdOx1 | Symptomatic | 14d | 87.0 (47.0–97.0) | Alpha |

Abbreviations: VE, vaccine effectiveness; HCWs, healthcare workers; GP, general population; Day_F, days after the full vaccination; RCT, randomized controlled trial; CI, confidence interval.

^a^ Median age (interquartile range)

^b^ Cases

**Table S5. VE of COVID-19 vaccines against B.1.351 (Beta) and P.1 (Gamma) variant**

| First author | Study design | Participants, N | Participants | Age  (years) | Vaccine | Outcomes | Day_F | VE% (95% CI) | Variants |
| --- | --- | --- | --- | --- | --- | --- | --- | --- | --- |
| Shinde (2021) | RCT | 4,387 | GP | 18-84 | NVX-CoV2373 | Symptomatic | 7d | 51.1 (-0.6–76.2) | Beta |
| Madhi (2021) | RCT | 750/714 | GP | 18-65 | ChAdOx1 | Symptomatic | 14d | 10.4 (-76.8–54.8) | Beta |
| Sadoff (2021) | RCT | 4,969 | GP | ≥18 | Ad26.COV2.S | Symptomatic | 14d | 38.1 (4.2–60.4) | Beta |
| Thomas (2021) | RCT | 800 | GP | ≥16 | BNT162b2 | All infections | 7d | 100.0 (53.5–100.0) | Beta |
| Abu-Raddad (2021) | Case-control | 35,979 ^b^ | GP | 33 (22-40) ^a^ | BNT162b2 | All infections | 14d | 75.0 (70.5–78.9) | Beta |
| Chemaitelly_1 (2021) | Case-control | 66,042 ^b^ | GP | 32 (25-39) ^a^ | mRNA-1273 | All infections | 14d | 96.4 (91.9–98.7) | Beta |
| Charmet (2021) | Case-control | 33,863 ^b^ | GP | ≥20 | BNT162b2/mRNA-1273 | All infections | 7d | 77.0 (63.0–86.0) | Beta |
| Tang (2021) | Case-control | 2,934 ^b^ | GP | 27 (12–36) ^a^ | BNT162b2/mRNA-1273 | All infections | 14d | 76.4 (72.9–79.4) | Beta |
|  |  |  | GP | 27 (12–36) ^a^ | BNT162b2 | All infections | 14d | 74.3 (70.3–77.7) | Beta |
|  |  |  | GP | 27 (12–36) ^a^ | mRNA-1273 | All infections | 14d | 80.8 (69.0–88.2) | Beta |
| Chemaitelly_2 (2021) | Case-control | 113,830 ^b^ | GP | 31 (21-39) ^a^ | BNT162b2 | All infections | 0-30d | 74.3 (67.9–79.5) | Beta |
| Sadoff (2021) | RCT | 4,969 | GP | ≥18 | Ad26.COV2.S | Symptomatic | 14d | 36.4 (13.9–53.2) | Gamma |
| Clemens (2021) | RCT | 10,416 | GP | ≥18 | ChAdOx1 | Symptomatic | 14d | 63.6 (-2.1–87.0) | Gamma |
| Bravo (2022) | RCT | 30,174 | GP | ≥18 | SCB-2019 | All infections | 14d | 91.8 (44.9–99.8) | Gamma |
| Kremsner (2022) | RCT | 39,680 | GP | ≥18 | CVnCoV | Symptomatic | 14d | 67.1 (29.8–84.6) | Gamma |
| Williams (2021) | Cohort | 143 | Residents of LTCH | NR | mRNA-1273 | All infections | 14d | 52.5 (26.9–69.1) | Gamma |
|  |  |  | Staff of LTCH | NR | mRNA-1273 | All infections | 14d | 66.2 (2.3–88.3) | Gamma |
| Nasreen (2022) | Case-control | 51,440 ^b^ | GP | ≥16 | BNT162b2 | Symptomatic | 14d | 85.0 (70.0–93.0) | Beta/Gamma |
|  |  |  | GP | ≥16 | BNT162b2 | Symptomatic | 14d | 90.0 (76.0–96.0) | Gamma |
|  |  |  | GP | ≥16 | BNT162b2 | Symptomatic | 14d | 86.0 (0.0–98.0) | Beta |
| Ranzani (2021) | Case-control | 43,774 ^b^ | Older | ≥70 | CoronaVac | Symptomatic | 14d | 46.8 (38.7–53.8) | Gamma |
| Hitchings_1 (2021) | Case-control | 418 ^b^ | HCWs | ≥18 | CoronaVac | Symptomatic | 14d | 36.8 (-54.9–74.2) | Gamma |
| Hitchings_2 (2021) | Case-control | 30,680 ^b^ | Older | ≥60 | ChAdOx1 | Symptomatic | 14d | 77.9 (69.2–84.2) | Gamma |
| Chung (2021) | Case-control | 324,033 ^b^ | GP | ≥16 | BNT162b2/mRNA-1273 | Symptomatic | 7d | 88.0 (61.0–96.0) | Beta/Gamma |
| Nasreen (2022) | Case-control | 51,440 ^b^ | GP | ≥16 | mRNA-1273 | Symptomatic | 14d | 88.0 (10.0–98.0) | Beta/Gamma |

Abbreviations: VE, vaccine effectiveness; HCWs, healthcare workers; LTCH, long term care homes; GP, general population; Day_F, days after the full vaccination; RCT, randomized controlled trial; CI, confidence interval; NR, not reported.

^a^ Median age (interquartile range)

^b^ Cases

**Table S6. VE of COVID-19 vaccines against B.1.617.2 (Delta) variant**

| First author | Study design | Participants, N | Participants | Age  (years) | Vaccine | Outcomes | VE% (95% CI) | Variant |
| --- | --- | --- | --- | --- | --- | --- | --- | --- |
| Sadoff (2022) | RCT | 4,969 | GP | ≥18 | Ad26.COV2.S_14d | Symptomatic | −6.0 (−178.3–59.2) | Delta |
| Ella (2021) | RCT | 16,973 | GP | 18-98 | BBV152_14d | Symptomatic | 65.2 (33.1–83.0) | Delta |
| Bravo (2022) | RCT | 30,174 | GP | ≥18 | SCB-2019_14d | All infections | 78.7 (57.3–90.4) | Delta |
| Lopez Bernal_1 (2021) | Case-control | 19,109 ^b^ | GP | ≥16 | BNT162b2/ChAdOx1_14d | Symptomatic | 79.6 (76.7–82.1) | Delta |
|  |  |  | GP | ≥16 | BNT162b2_14d | Symptomatic | 88.0 (85.3–90.1) | Delta |
|  |  |  | GP | ≥16 | ChAdOx1_14d | Symptomatic | 67.0 (61.3–71.8) | Delta |
| Li (2021) | Case-control | 74 ^b^ | GP | 18-59 | CoronaVac/CNBG_14d | All infections | 59.0 (16.0–81.6) | Delta |
| Nanduri (2021) | Cohort | 5,965,607 | Older | NR | BNT162b2/mRNA-1273_14d | All infections | 53.1 (49.1–56.7) | Delta |
|  |  |  | Older | NR | BNT162b2_14d | All infections | 52.4 (48.0–56.4) | Delta |
|  |  |  | Older | NR | mRNA-1273_14d | All infections | 50.6 (45.0–55.7) | Delta |
|  |  |  | HCWs | NR | mRNA-1273/BNT162b2_14d | All infections | 66.0 (26.0–84.0) | Delta |
| Pouwels (2021) | Cohort | 743,526 | GP | 18-64 | BNT162b2_14d | All infections | 80.0 (77.0–83.0) | Delta (>50%) |
|  |  |  | GP | 18-64 | ChAdOx1_14d | All infections | 67.0 (62.0–71.0) | Delta (>50%) |
|  |  |  | GP | 18-64 | BNT162b2_14d | Symptomatic | 84.0 (82.0–86.0) | Delta (>50%) |
|  |  |  | GP | 18-64 | ChAdOx1_14d | Symptomatic | 71.0 (66.0–74.0) | Delta (>50%) |
| Glatman-Freedman (2021) | Cohort | 601,625 | Adolescents | 12-15 | BNT162b2_8-28d | All infections | 91.5 (88.2–93.9) | Delta |
| Seppälä (2021) | Cohort | 18,431 | GP | ≥18 | BNT162b2/mRNA-1273_7d | All infections | 64.6 (60.6–68.2) | Delta |
| Fabiani (2022) | Cohort | 33,250,344 | GP | ≥16 | BNT162b2/mRNA-1273_14d | All infections | 69.0 (66.7–71.2) | Delta |
|  |  |  | Older | 60-79 | BNT162b2/mRNA-1273_14d | All infections | 56.4 (51.2–61.0) | Delta |
|  |  |  | Older | ≥80 | BNT162b2/mRNA-1273_14d | All infections | 35.0 (17.4–48.9) | Delta |
| Fabiani (2022) | Cohort |  | HCWs | NR | BNT162b2/mRNA-1273_14d | All infections | 54.0 (39.1–65.2) | Delta |
| Risk (2022) | Cohort | 159,055 | GP | ≥18 | Ad26.COV2.S_14d | All infections | 50.0 (31.0–63.0) | Delta (>50%) |
|  |  |  | GP | ≥18 | BNT162b2_14d | All infections | 53.0 (47.0–58.0) | Delta |
|  |  |  | GP | ≥18 | mRNA-1273_14d | All infections | 70.0 (64.0–75.0) | Delta |
| Kang (2022) | Cohort | 10,805 | GP | ≥18 | CoronaVac/HB02_14d | All infections | 51.8 (20.3–83.2) | Delta |
|  |  |  | GP | ≥18 | CoronaVac/HB02_14d | Symptomatic | 60.4 (31.8–88.9) | Delta |
| Poukka (2022) | Cohort | 427,905 | HCWs | 16-69 | BNT162b2/mRNA-1273_14-90d | All infections | 85.0 (81.0–88.0) | Delta |
|  |  |  | HCWs | 16-69 | ChAdOx1_14-90d | All infections | 88.0 (71.0–95.0) | Delta |
| Wu (2022) | Cohort | 1,462 | Close contacts | ≥18 | BBIBP-CorV_14d | Symptomatic | 50.5 (3.8–74.6) | Delta |
|  |  |  | Close contacts | ≥18 | CoronaVac_14d | Symptomatic | 39.1 (-0.9–63.3) | Delta |
| Lutrick (2021) | Cohort | 243 | Adolescents | 12-17 | BNT162b2_14d | All infections | 92.0 (79.0–97.0) | Delta |
| Reis (2021) | Cohort | 188,708 | Adolescents | 12-18 | BNT162b2_7-21d | All infections | 90.0 (88.0–92.0) | Delta |
|  |  |  | Adolescents | 12-18 | BNT162b2_7-21d | Symptomatic | 93.0 (88.0–97.0) | Delta |
| Tartof (2021) | Cohort | 3,436,957 | GP | ≥12 | mRNA-1273_7-36d | All infections | 93.0 (85.0–97.0) | Delta |
| Sheikh (2021) | Case-control | 19,543 ^b^ | GP | ≥16 | BNT162b2_14d | All infections | 79.0 (75.0–82.0) | Delta |
|  |  |  | GP | ≥16 | ChAdOx1_14d | All infections | 60.0 (53.0–66.0) | Delta |
| Chemaitelly_2 (2021) | Case-control | 113,830 ^b^ | GP | 31 (21-39) ^a^ | BNT162b2_0-30d | All infections | 87.6 (79.7–92.3) | Delta |
| Nasreen (2022) | Case-control | 51,440 ^b^ | GP | ≥16 | BNT162b2_14d | Symptomatic | 92.0 (89.0–94.0) | Delta |
|  |  |  | GP | ≥16 | mRNA-1273_14d | Symptomatic | 94.0 (90.0–97.0) | Delta |
|  |  |  | GP | ≥16 | ChAdOx1_14d | Symptomatic | 88.0 (68.0–96.0) | Delta |
| Tang (2021) | Case-control | 2,934 ^b^ | GP | 27 (12–36) ^a^ | BNT162b2/mRNA-1273_14d | All infections | 55.5 (51.2–59.4) | Delta |
|  |  |  | GP | 27 (12–36) ^a^ | BNT162b2_14d | All infections | 51.9 (47.0–56.4) | Delta |
|  |  |  | GP | 27 (12–36) ^a^ | mRNA-1273_14d | All infections | 73.1 (67.5–77.8) | Delta |
|  |  |  | GP | 27 (12–36) ^a^ | BNT162b2/mRNA-1273_14d | Symptomatic | 49.2 (42.8–54.9) | Delta |
|  |  |  | GP | 27 (12–36) ^a^ | BNT162b2_14d | Symptomatic | 44.4 (37.0–50.9) | Delta |
|  |  |  | GP | 27 (12–36) ^a^ | mRNA-1273_14d | Symptomatic | 73.9 (65.9–79.9) | Delta |
| Grannis (2021) | Case-control | 3,657 ^b^ | GP | ≥18 | BNT162b2/mRNA-1273/Ad26.COV2.S_14d | Symptomatic | 82.0 (81.0–84.0) | Delta (>50%) |
|  |  |  | GP | ≥18 | BNT162b2_14d | Symptomatic | 77.0 (74.0–80.0) | Delta (>50%) |
|  |  |  | GP | ≥18 | mRNA-1273_14d | Symptomatic | 92.0 (89.0–93.0) | Delta (>50%) |
|  |  |  | GP | ≥18 | Ad26.COV2.S_14d | Symptomatic | 65.0 (56.0–72.0) | Delta (>50%) |
| Sritipsukho (2022) | Case-control | 1,118 ^b^ | GP | ≥18 | CoronaVac/ChAdOx1_14d | All infections | 65.0 (56.0–72.0) | Delta (>95%) |
|  |  |  | GP | ≥18 | CoronaVac_14d | All infections | 60.0 (49.0–69.0) | Delta |
|  |  |  | GP | ≥18 | ChAdOx1_14d | All infections | 83.0 (70.0–90.0) | Delta |
| Klein (2022) | Case-control | 3,860 ^b^ | GP | ≥18 | BNT162b2/mRNA-1273_14-179d | Symptomatic | 86.0 (85.0–87.0) | Delta (>50%) |
| Oliveira (2022) | Case-control | 186 ^b^ | Adolescents | 12-18 | BNT162b2_14d | All infections | 90.0 (79.0–95.0) | Delta (>92%) |
|  |  |  | Adolescents | 12-18 | BNT162b2_14d | Symptomatic | 93.0 (81.0–97.0) | Delta (>92%) |
|  |  |  | Adolescents | 12-18 | BNT162b2_14d | Asymptomatic | 85.0 (57.0–95.0) | Delta (>92%) |
| Tseng (2022) | Case-control | 23,512 ^b^ | GP | ≥18 | mRNA-1273_14-90d | All infections | 80.2 (68.2–87.7) | Delta |
|  |  |  | GP | ≥18 | **mRNA-1273_3D_14-60d** ^c^ | All infections | 92.0 (91.0–94.0) | Delta |
| Ferdinands (2022) | Case-control | 18, 637 ^b^ | GP | ≥18 | BNT162b2/mRNA-1273_0-60d | Symptomatic | 84.0 (83.0–84.0) | Delta (>50%) |
|  |  |  | GP | ≥18 | **BNT162b2/mRNA-1273_3D_0-60d** ^c^ | Symptomatic | 90.0 (89.0–90.0) | Delta (>50%) |
| Britton (2022) | Case-control | 329,057 ^b^ | GP | ≥20 | BNT162b2_14-60d | Symptomatic | 38.0 (35.0–42.0) | Delta |
|  |  |  | GP | ≥20 | mRNA-1273_14-60d | Symptomatic | 90.0 (89.0–91.0) | Delta |
|  |  |  | GP | ≥20 | Ad26.COV2.S_14-60d | Symptomatic | 94.0 (92.0–96.0) | Delta |
|  |  |  | Adolescents | 16-19 | BNT162b2_14-60d | Symptomatic | 54.0 (38.0–70.0) | Delta |
|  |  |  | Adolescents | 16-19 | mRNA-1273_14-60d | Symptomatic | 67.0 (63.0–71.0) | Delta |
|  |  |  | Adolescents | 16-19 | Ad26.COV2.S_14-60d | Symptomatic | 67.6 (67.3–67.9) | Delta |
| Grant (2022) | Case-control | 8,644 ^b^ | GP | ≥20 | BNT162b2/mRNA-1273_7d | Symptomatic | 89.7 (89.5–89.8) | Delta |
| Andrews_1 (2022) | Case-control | 1,125,257 ^b^ | GP | ≥16 | ChAdOx1-S_14-63d | Symptomatic | 59.1 (55.4–62.6) | Delta |
|  |  |  | GP | ≥16 | BNT162b2_14-63d | Symptomatic | 79.6 (77.0–81.8) | Delta |
|  |  |  | Older | ≥65 | ChAdOx1-S_14-63d | Symptomatic | 63.1 (51.5–72.1) | Delta |
|  |  |  | Older | ≥65 | BNT162b2_14-63d | Symptomatic | 93.7 (92.2–94.9) | Delta |
| Thiruvengadam (2021) | Case-control | 2,766 ^b^ | GP | 35 (28–45) ^a^ | ChAdOx1_14d | Symptomatic | 97.0 (96.0–97.0) | Delta |
| Andrews_2 (2022) | Case-control | 343,955 ^b^ | GP | 18-49 | **ChAdOx1-S_2D/BNT162b2_B_14-35d** ^c^ | Symptomatic | 94.3 (93.8–94.8) | Delta |
|  |  |  | GP | 18-49 | **ChAdOx1-S_2D/mRNA-1273_B_14-35d** ^c^ | Symptomatic | 97.4 (95.5–98.5) | Delta |
|  |  |  | GP | 18-49 | **BNT162b2_3D_14-35d** ^c^ | Symptomatic | 93.9 (93.6–94.2) | Delta |
|  |  |  | GP | 18-49 | **BNT162b2_2D/mRNA-1273_14-35d** ^c^ | Symptomatic | 96.7 (94.4–98.0) | Delta |
|  |  |  | Older | ≥50 | **ChAdOx1-S_2D/BNT162b2_B_14-35d** ^c^ | Symptomatic | 94.4 (94.1–94.7) | Delta |
|  |  |  | Older | ≥50 | **ChAdOx1-S_2D/mRNA-1273_B_14-35d** ^c^ | Symptomatic | 97.0 (96.0–97.8) | Delta |
|  |  |  | Older | ≥50 | **BNT162b2_3D_14-35d** ^c^ | Symptomatic | 94.4 (94.1–94.7) | Delta |
|  |  |  | Older | ≥50 | **BNT162b2_2D/mRNA-1273_14-35d** ^c^ | Symptomatic | 94.8 (92.7–96.3) | Delta |

Abbreviations: VE, vaccine effectiveness; HCWs, healthcare workers; GP, general population; RCT, randomized controlled trial; CI, confidence interval; NR, not reported.

^a^ Median age (interquartile range)

^b^ Cases

^c^ Booster vaccination

**Table S7. VE of COVID-19 vaccines against B.1.1.529 (Omicron) variant**

| First author | Study design | Participants, N | Participants | Age  (years) | Vaccine | Outcomes | VE% (95% CI) | Variant |
| --- | --- | --- | --- | --- | --- | --- | --- | --- |
| Tseng (2022) | Case-control | 23,512 ^b^ | GP | ≥18 | mRNA-1273_14-90d | All infections | 44.0 (35.1–51.6) | Omicron |
|  |  |  | GP | ≥18 | **mRNA-1273_3D_14-60d** ^a^ | All infections | 71.6 (69.7–73.4) | Omicron |
| Ferdinands (2022) | Case-control | 18, 637 ^b^ | GP | ≥18 | BNT162b2/mRNA-1273_0-60d | Symptomatic | 69.0 (62.0–75.0) | Omicron |
|  |  |  | GP | ≥18 | **BNT162b2/mRNA-1273_3D_0-60d** ^a^ | Symptomatic | 87.0 (85.0–88.0) | Omicron |
| Klein (2022) | Case-control | 3,860 ^b^ | GP | ≥18 | **BNT162b2/mRNA-1273_14-179d** ^a^ | Symptomatic | 52.0 (46.0–58.0) | Omicron |

Abbreviations: VE, vaccine effectiveness; GP, general population; CI, confidence interval.

^a^ Booster vaccination

^b^ Cases
